# War Work of the American Medical Women

**Published:** 1917-11

**Authors:** Eliza M. Mosher


### Journal-Record of Medicine

Successor to Atlanta Medical and Surgical Journal, Established 1855
and Southern Medical Record, Established 1870

**OWNED BY THE ATLANTA MEDICAL JOURNAL COMPANY**
PUBLISHED MONTHLY

*Official Organ Fulton County Medical Society, State Examing Board, Atlanta, Birmingham and Atlantic Railroad Surgeon's Association, Chattahoochee Valley Medical and Surgical Association, Etc.*

A. W. STIRLING, M. D., C. M., D. P. H.; J. S. HURT, B. Ph., M.D.
GEO. M. NILES, M. D., W. J. LOVE, M. D., (Ala.); Associate Editors

E. W. ALLEN, Business Manager

**COLLABORATORS**

W. F. WESTMORELAND, M. D., General Surgery
F. W. McRAE, M. D., Abdominal Surgery
ALLEN H. BUNCE, Medical Societies
E. B. BLOCK, M. D., Diseases of the Nervous System
MICHAEL HOKE, M. D., Orthopedic Surgery
CYRUS W. STRICKLER, M. D., Legal Medicine and Medical Legislation
E. C. DAVIS, A. B., M. D., Obstetrics
E. G. JONES, A. B., M. D., Gynecology
ALLEN H. BUNCE, M. D., Pathology and Bacteriology
R. T. DORSEY, JR., B. S., M. D., Medicine
L. M. GAINES, A. B., M. D., Internal Medicine
GEO. C. MIZELL, M. D., Diseases of the Stomach and Intestines
L. B. CLARKE, M. D., Pediatrics
EDGAR PAULLIN, M. D., Opsonic Medicine
THEODORE TOEPEL, M. D., Mechano Therapy
E. G. BALLENGER, M. D., Diseases of the Genito-Urinary Organs

VOL. LXIV. ATLANTA, GA., NOV., 1917. NO. 8

### WAR WORK OF THE AMERICAN MEDICAL WOMEN.*

**By Eliza M. Mosher, M.D.**

The second annual meeting of the Medical Women's National Association, Dr. Bertha Van Hoosen of Chicago, President, was held in New York City, June, 1917. In view of the pressing need of physicians and surgeons in the war zone and in the devastated districst of Europe, a War Service Committee was appointed by the Association to deal with the situation. This body created an Executive Committee with defined powers, of which Dr. Rosalie Slaughter Morton was unanimously elected Chairman, Dr. Morton's selection for this post was a wise one. The Serbian Government has bestowed upon her a decoration for her service in that country. In France special privileges had been given her to inspect and study the French hospitals, and after returning home from foreign duty she has still kept in close touch with the work.

Mr. Leo Schlesinger, of New York City, places at the disposal of the Committee a suite of rooms in his office building, 637 Madison Avenue, admirably suited to its purpose, and there early in June the Committee was installed and intensive work began. Before the Committee had completed its organization, Dr. Franklin Martin, Chairman of the General Medical Board of Washington, asked for an outline of its plan of work. This outline which Dr. Morton presented in person, received the unanimous approval of the Board and Dr. Morton was appointed a member of it and Chairman of a committee of nine women physicians from different parts of the country, who were selected from a list of twelve submitted to Dr. Martin.

This Committee of Women Physicians of the General Medical Board, may be regarded in the light of a congressional committee, its constituency being the women physicians of the United States. If the latter wish to have force and efficiency, organization is necessary. This committee of nine members is not permitted to increase the membership of the General Medical Board; obviously, therefore, it could not encompass the extensive work now going forward under the American Woman's Hospitals, which it is hoped, the general co-operation of the women throughout the country will make even more extensive and thorough, and consequently of more value to the General Board. We are now in a position to supply the data necessary to supplement that on the cards sent out from Washington, and on file there.

Copies of the outline prepared for the General Medical Board were laid before Col. J. R. Kean, Director of the Department of Military Relief of the American Red Cross and the Surgeon General of the Army, General Gorgas. They both expressed the greatest interest in the approval of the work, General Gorgas said that if the war continued for any length of time, the services of every woman doctor in the country would doubtless eventually be needed.

To anticipate this need, the plan of work, with registration blanks, was mailed to 1,000 medical women, asking them to enroll. On October 6th, at the time the first quarterly report of the American Woman's Hospitals was issued, 115 women had registered as follows:

1. Women's Units, 150; 11. Women's Units to Allies' armies, 110; 111. Service in established Units, 103; IV. Maternity Units to devastated regions, 84; V. Village practice, 25; VI. For service in any of the above five, without choice, 110. The registration blanks are still coming in and it is hoped that every woman physician in the country will record herself as being willing to serve her country in its hour of need.

Dr. Ester Lovejoy of Portland, Oregon, and Dr. Alice Barlow, of Winnetka, Ill., are now making a study of Civilian conditions for our War Service Committee and the following doctors, members of the American Women's Hospitals have been sent by the Red Cross to the other side:

Esther L. Blair, M.D., Pittsburg, Pa.
Women's Medical College of Pa.
Dorothy Child, M.D.,
John Hopkins University.
Florence Child, M.D.,
John Hopkins University.
Edith Lyon Heard, M.D.,
Women's Medical College, Pa.
Mary Nevin, M.D.
Esther E. Parker, M.D.,
Cornell University.
Helen L. H. Woodroffe, M.D.,
Denver Homeopathic, 1900.

Marion C. Stevens, D.D.S.,
Tufts College.
Ira R. Shields, M.D.,
University of London, England.
Laura C. Wiggins,
Anaesthetist.

In September the Red Cross asked for two units of women doctors to go immediately to Roumania. Their departure has been delayed for diplomatic reasons, incident to the situation in Russia. There are also in readiness forty doctors, who may be called within the next thirty days, and units have been arranged which can be mobilized within a few hours.

The women doctors present an attractive appearance in their uniforms, which were planned by Dr. Morton at the request of the Red Cross. The lines of the Red Cross uniform for men are follewed, and the uniform is both smart and attractive.

The American Women's Hospitals' flag and proper insignia designed by Miss Brenda Putnam, a niece of that brilliant pioneer among women physicians, the late Mary Putnam Jacobi, has been adopted. The flag is blue and white; the drooping wings, the symbols of the American Women's Hospitals, are grouped around a shield bearing the name "American Women's Hospitals." The pins of bronze, are sheltering wings, denoting protection and comfort, with the emblem of the various branches of the service placed upon them.

Open meetings of the American Women's Hospitals were held every Thursday afternoon throughout the past summer and will be continued indefinitely. These meetings presided over by Dr. Morton, or in her absence by Dr. Emily Dunning Barringer, the Vice Chairman, have been of great interest. not only to the members of the organization, but to the general public.

Inspiring speeches by friends of the organization, and officers, doctors and nurses returned from the front have been a feature of these meetings. One of the most interesting of these was the address made by H. Liebert, the French Consul General at New York.

An important branch of the American Women's Hospitals is that of the A. V. A. (American Volunteer Aid). This body was formed after the British V. A. D. (Volunteer Aid Department) and is in a thriving condition. Those wishing to join are given forms on which must be entered all data concerning non-medican women who wish to be laboratory assistants, ambulance drivers, stretcher bearers, interpreters, dieticians, clerks, etc. A number will be needed in the units already in readiness. These lay assistants have a distinctive uniform for both identification and protection.

The Surgeon General of the Army, has expressed his willingness to place in base hospitals, as Contract-Surgeons, women physicians as anaethestists, radiographers, and laboratory workers at a salary to be arranged by contract, and not to exceed $1,800 per year. The need for laboratory workers is so great that the American Women's Hospitals have opened courses in this branch at the Women's Medical College of Pennsylvania; Women's Hospitals, New York; and at the Research Laboratories of the New York City Board of Health. In them courses will be given to college women who have already studied chemistry and biology, in order to fit them, at a nominal expense, to become laboratory technicians, and to assist our physicians.

Any physician connected with laboratories which offer such courses in the different parts of the United States, and when wishing to apply for this training are requested to take up the matter immediately with the National Chairman of Laboratory work, Dr. Martha Wollenstein, No. 1 West 81st Street, New York City.

The chairman of the Committee on Army Hospitals in the Home Zone, both for acute and convalescent cases, is Dr. Mary Almira Smith, 33 Newbury Street, Boston, Mass. The American Women's Hospitals, have in Boston, two hospitals in readiness for convalescent cases and several others near

New York. Its Women's Army General Hospital of New York, which has recorded its personnel and equipment in the War Department at Washington, has been told by Surgeon-General Gorgas, that it will be notified when this is needed, and that it has the same status as all other army hosptials in the Home Zone.

The Women's Committee of the General Medical Board has had two meetings, July 29th and September 29. A registration card was sent to the women physicians of the United States with a view to ascertaining how many would be willing to serve in base hospitals as contract-surgeons, radiographers, laboratory workers and dressers of wounds. These cards are now being filed in Washington for reference in case need arises to place women in base hospitals to release men for field hospital service.

The following are the regulations regarding contract practice:

1. Contract-Surgeons do not receive pensions except by special act of Congress.

2. The government pays for transportation, quarters, heat and light, the same as furnished the first lieutenants.

3. There is no additional pay for foreign service; the contract specifies where the service is to be to the amount to be received for the special service.

4. $1.800 a year is the maximum, the minimum being whatever agreed to for the particular service to be rendered.

5. The amount is regulated by agreement; the surgeon states his price and the government accepts or rejects; or vice versa.

6. The immediate superiors are the commissioned officers of whatever in command at the station where the contract-surgeon services, even although they be only first lieutenants.

7. The Surgeon-General's office expressed an interest in knowing how many women wished to become members of the Army Reserve Corps, and a letter was sent by the General Medical Board Committee of Women Physicians to the presidents of Memical Women's organizations asking an expression of preference for this service, but comparatively few made their offer of war service absolutely contingent upon their becoming officers in the Army Reserve Corps.

It is the intention of the Medical Women's National Association to continue the work of this War Service Committee until the end of the war if the need for it continues to exist.

